# Alterations of the gut microbial community structure and function with aging in the spontaneously hypertensive stroke prone rat

**DOI:** 10.1038/s41598-022-12578-7

**Published:** 2022-05-20

**Authors:** Huanan Shi, James W. Nelson, Sharon Phillips, Joseph F. Petrosino, Robert M. Bryan, David J. Durgan

**Affiliations:** 1 Department of Anesthesiology, Baylor College of Medicine, Houston, TX 77030 USA; 2 Integrative Physiology, Baylor College of Medicine, Houston, TX 77030 USA; 3 Molecular Virology and Microbiology, Baylor College of Medicine, Houston, TX 77030 USA; 4 Integrated Molecular and Biomedical Sciences Graduate Program, Baylor College of Medicine, Houston, TX 77030 USA; 5 The Alkek Center for Metagenomics and Microbiota Research, Baylor College of Medicine, Houston, TX 77030 USA

**Keywords:** Physiology, Cardiology

## Abstract

Gut dysbiosis, a pathological imbalance of bacteria, has been shown to contribute to the development of hypertension (HT), systemic- and neuro-inflammation, and blood–brain barrier (BBB) disruption in spontaneously hypertensive stroke prone rats (SHRSP). However, to date individual species that contribute to HT in the SHRSP model have not been identified. One potential reason, is that nearly all studies of the SHRSP gut microbiota have analyzed samples from rats with established HT. The goal of this study was to examine the SHRSP gut microbiota before, during, and after the onset of hypertension, and in normotensive WKY control rats over the same age range. We hypothesized that we could identify key microbes involved in the development of HT by comparing WKY and SHRSP microbiota during the pre-hypertensive state and longitudinally. Systolic blood pressure (SBP) was measured by tail-cuff plethysmography and fecal microbiota analyzed by16S rRNA gene sequencing. SHRSP showed significant elevations in SBP, as compared to WKY, beginning at 8 weeks of age (*p* < 0.05 at each time point). Bacterial community structure was significantly different between WKY and SHRSP as early as 4 weeks of age, and remained different throughout the study (*p* = 0.001–0.01). At the phylum level we observed significantly reduced Firmicutes and Deferribacterota, and elevated Bacteroidota, Verrucomicrobiota, and Proteobacteria, in pre-hypertensive SHRSP, as compared to WKY. At the genus level we identified 18 bacteria whose relative abundance was significantly different in SHRSP versus WKY at the pre-hypertensive ages of 4 or 6 weeks. In an attempt to further refine bacterial candidates that might contribute to the SHRSP phenotype, we compared the functional capacity of WKY versus SHRSP microbial communities. We identified significant differences in amino acid metabolism. Using untargeted metabolomics we found significant reductions in metabolites of the tryptophan-kynurenine pathway and increased indole metabolites in SHRSP versus WKY plasma. Overall, we provide further evidence that gut dysbiosis contributes to hypertension in the SHRSP model, and suggest for the first time the potential involvement of tryptophan metabolizing microbes.

Under normal physiological conditions, commensal gut microbes are beneficial to the host by synthesizing important metabolites and vitamins, metabolizing indigestible carbohydrates, protecting against invading pathogens, maintaining a healthy immune system, and more^1–3^. Conversely, when the balance of bacteria in the gut is pathologically altered, a state referred to as dysbiosis, the microbiota can have detrimental effects not only in the gut but also in organs distant to the gut. These detrimental effects distant to the gut can be observed in metabolic diseases, cardiovascular diseases, neurodegenerative diseases, and behavioral and neurological disorders^1,4–6^.

Recently, we and others have demonstrated that gut dysbiosis in spontaneously hypertensive rats (SHRs) and spontaneously hypertensive stroke-prone rats (SHRSPs) has an underlying role in hypertension, neural and systemic inflammation, and disruption of the blood–brain barrier (BBB) when compared to control Wistar–Kyoto rats (WKYs)^7–13^. At the present time we know very little about the bacterial genera or the bacterial mechanisms that elicits hypertension in this model. However, two potential candidates recently reported include bile acids^13^ and short chain fatty acids^14^. Gut bacteria can generate short chain fatty acids by fermentation^3^, and metabolize bile acids generated by the host^15^.

In the present study, we tested the hypothesis that we could identify key microbes involved in the development of hypertension (HT) by comparing WKY and SHRSP microbiota during the pre-hypertensive state and longitudinally. We demonstrate significantly different gut microbiota communities between WKY and SHRSP from 4 to 20 weeks old. We observed a number of “candidate” genera whose relative abundance was significantly different between WKY and SHRSP at ages when hypertension was developing in SHRSP. Seeing as that a single genus could not be identified, we assessed the functional capacity of the community as a whole and identified that a number of genera significantly different between WKY and SHRSP are involved in short chain fatty acid and amino acid metabolism. In support of this, we found significant reductions in the short chain fatty acids propionate and butyrate, and reduced tryptophan-kynurenine pathway metabolites, in SHRSP versus WKY plasma. Not only do these data support previous findings that gut dysbiosis contributes to the SHRSP phenotype, they also show complex changes in the SHRSP microbiome with age, and demonstrate significant alterations in circulating microbial metabolites that may contribute to the development of HT.

## Methods

All animal protocols were approved by the Institutional Animal Care and Use Committee at Baylor College of Medicine, Houston, TX and conformed to the Guide for the Care and Use of Laboratory Animals, 8th edition, published by the National Institutes of Health (NIH). WKY and SHRSP were obtained from Charles Rivers and mated for at least four generations to produce in-house colonies for each strain. Rats of the same age and group were housed 2–4 per cage with ad libitum access to normal rodent chow (Labdiet 5V5R) and chlorinated water. Rats were housed in autoclaved cages with sterilized bedding (Biofresh pelleted cellulose), and were subjected to a 12 h light (6 AM– 6 PM): 12 h dark (6 PM–6 AM) cycle. Given the differences in the microbiota and inflammatory responses between male and females, gender must be treated in separate groups. We included only males in the present study since inclusion of both genders would be prohibitive for a single study.

### Studies with WKY and SHRSP

Male SHRSP and WKY rat pups were weaned at 21 days. Beginning at 4 weeks, fecal samples were collected for gut microbiota analysis and continued at intervals until the age of 20 weeks. Fecal pellets were collected for 16S rRNA analysis at 4, 6, 8, 10, 16, and 20 weeks, and systolic blood pressure (SBP) was measured as described every 2 weeks from 6 weeks until the age of 20 weeks. SBP was measured in unanesthetized rats using a six channel CODA high-throughput tail-cuff blood pressure system (Kent Scientific, Torrington, CT). Prior to the initial SBP measurement all rats were acclimatized to the system. At least 10 consecutive readings, without movement artifact, were averaged to obtain an individual measurement.

### Gut microbiota analysis

Fresh fecal pellets were collected into 1.5 mL tubes while handling rats, snap frozen, and stored at −80 °C. The samples were sent to the Center for Metagenomics and Microbiota Research (CMMR) at the Baylor College of Medicine where 16S rRNA gene sequence libraries were generated from the V4 primer region using the Illumina MiSeq platform^12,16,17^ after extracting DNA using MO BIO PowerMag Soil Isolation Kit (MO BIO Laboratories). Reads were de-noised and merged into amplicon sequence variants (ASVs) by DADA2 pipeline in R^18,19^. Taxonomic annotations were also generated against DADA2-formatted training FASTA files derived from SILVA138 Database^20^. ASVs with identical taxonomic assignment were grouped into taxonomic bins. 16S rRNA sequencing data is publicly available at GenBank under the accession KFUL00000000. ASVs were analyzed and visualized with ATIMA version 2 (Agile Toolkit for Incisive Microbial Analyses) developed by the CMMR at the Baylor College of Medicine.

### Microbial functional prediction

ASVs were input into PICRUSt2 (Phylogenetic Investigation of Communities by Reconstruction of Unobserved States) pipeline for functional prediction as previously described^21,22^. Stratified gene families with KEGG orthologs annotations were used to construct Gut-Brain Modules (GBM)^23^. Spearman's rank correlation coefficient was used to calculate correlation and statistical significance between taxa and GBM. Correlations with $\left|\rho \right|\ge 0.6$ and *p* < 0.05 were included.

### Untargeted metabolomics

Plasma was collected from 15-week-old rats in sterile tubes and snap frozen. Samples were submitted to Metabolon, Inc. (Morrisville, NC) for untargeted metabolomics. Plasma (100ul) samples (n = 6–8 per group) were subjected to methanol extraction. The purified supernatant was divided into aliquots corresponding to the various analytical methodologies, then subsequently evaporated and reconstituted with the appropriate analytical injection solvent. Samples were analyzed with four separate methods: two positive mode methods (Pos Early UHPLC-RP/MS/MS and Pos Late UHPLC-RP/MS/MS) and two negative mode methods (Neg UHPLC-RP/MS/MS and Neg UHPLC HILIC/MS/MS) to ensure broad coverage of biochemicals. Metabolites were identified by automated comparison of ion features to a reference library of chemical standards followed by visual inspection for quality control. For downstream analysis, any missing values were assumed to be below the limits of detection and were imputed with the compound minimum (minimum value imputation). Log 10 transformation was performed prior to statistical analysis. Random forest models for untargeted metabolomics were constructed as previously described^13^.

### Plasma SCFA measurements

Plasma was submitted to the Metabolomics Core at Baylor College of Medicine for SCFA measurement by HPLC as previously described^17^. Data was acquired and analyzed with Agilent MassHunter software.

### Statistical analysis

Data is expressed as means ± standard error of the mean or the standard error of least squares mean. *p* < 0.05 adjusted for multiple comparisons or false rate of discovery (FDR) was considered statistically significant. Data for taxa abundance were analyzed using the Mann–Whitney U test with a FDR adjusted for multiple comparisons. Two-way repeated measures ANOVA was used to analyze SBP with post hoc Holm-Sidak test where appropriate. β diversities were measured using weighted UniFrac, visualized by principal coordinate analysis (PCoA), and statistically analyzed using permutational multivariate analysis of variance (PERMANOVA).

## Results

**Figure 1:**
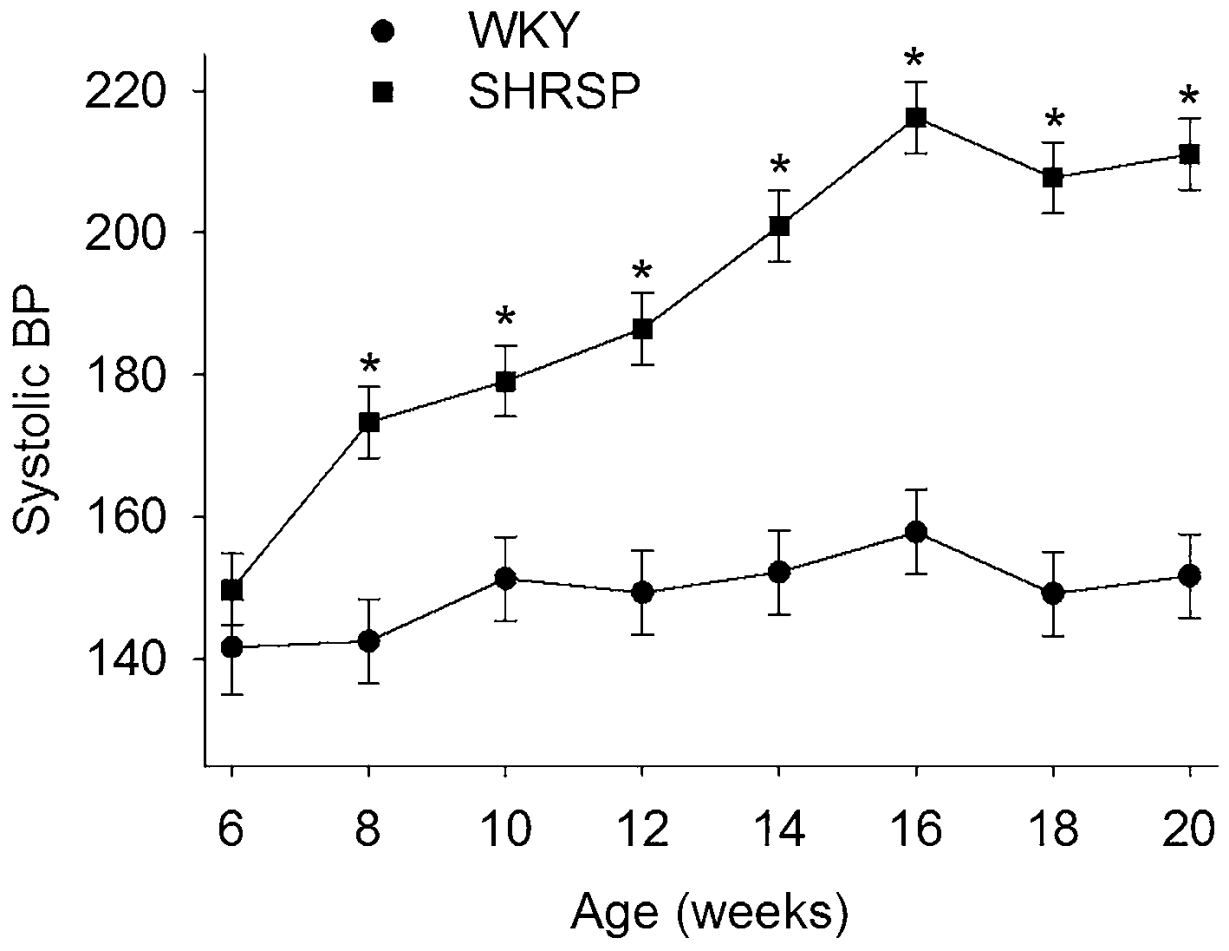
Systolic blood pressure (SBP) in WKY and SHRSP from 6 to 20 weeks of age. Two-way repeated measures ANOVA showed statistical main effects of strain, age, and interaction between strain and age (*p* < 0.001 for all, n = 5–7). *p ≤ 0.002 compared to corresponding age in WKYs (Holm-Sidak method).

We followed blood pressure in WKY and SHRSP from 6 to 20 weeks. Figure 1 shows SBP in SHRSPs began increasing between 6 and 8 weeks and continued to increase until 16 weeks when it plateaued above 200 mmHg. SBP in the WKYs remained between 140 and 150 mmHg at all ages.

**Figure 2:**
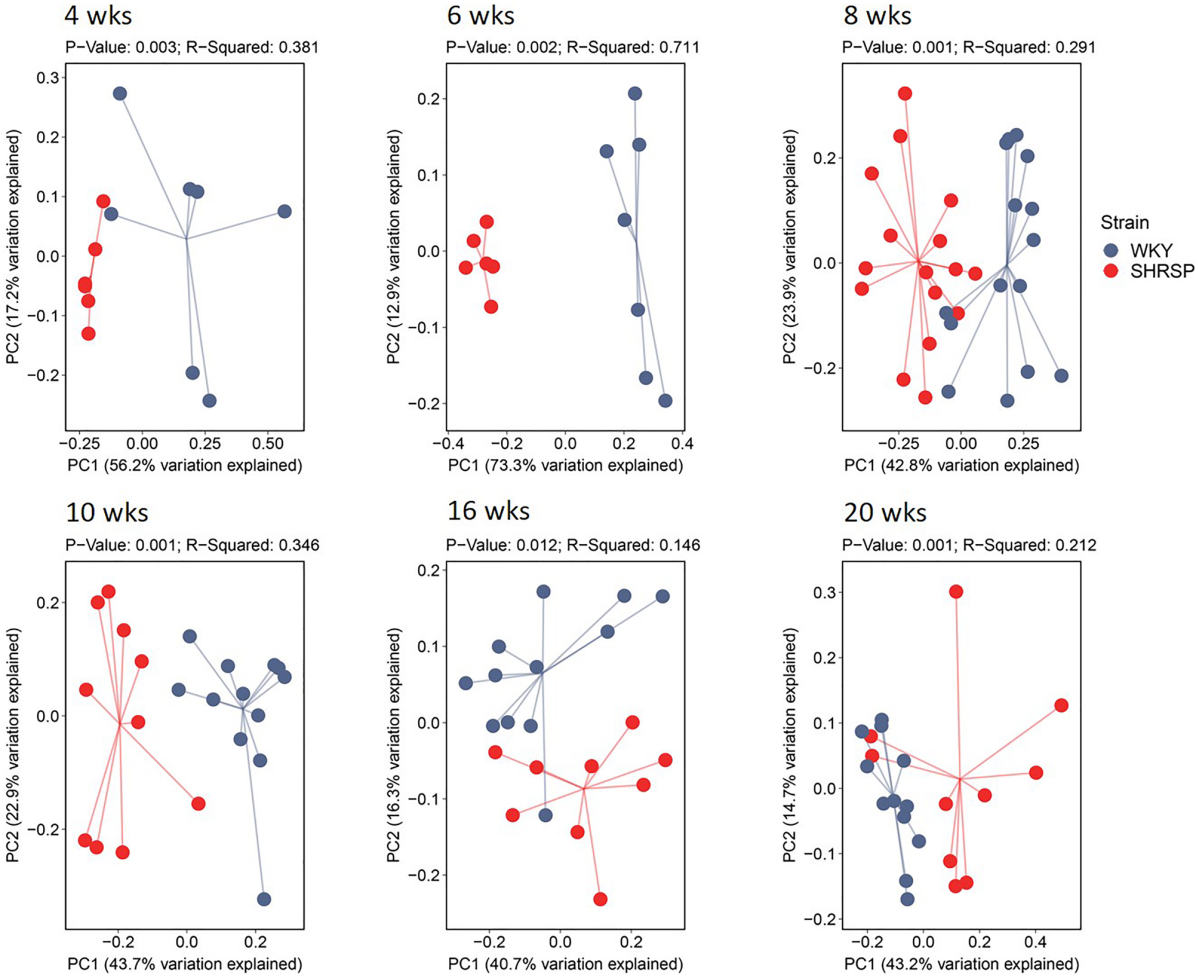
PCoAs of weighted UniFrac, measure of β diversities, comparing SHRSPs and WKYs at 4, 6, 8, 10, 16, and 20 weeks of age. The p-values, as determined using permutational multivariate analysis of variance (PERMANOVA), comparing SHRSPs and WKYs at each age group are presented at the top of each plot. n = 6–15.

At intervals, fresh fecal samples were obtained for analysis of bacterial abundance and community structure using 16S rRNA analysis. Figure 2 shows PCoA of weighted UniFrac distances, a measure of β diversity, comparing SHRSPs and WKYs at different ages. Bacterial community differences were highly significant between strains from 4 to 20 weeks of age (ranging from *p* = 0.001–0.01).

**Figure 3:**
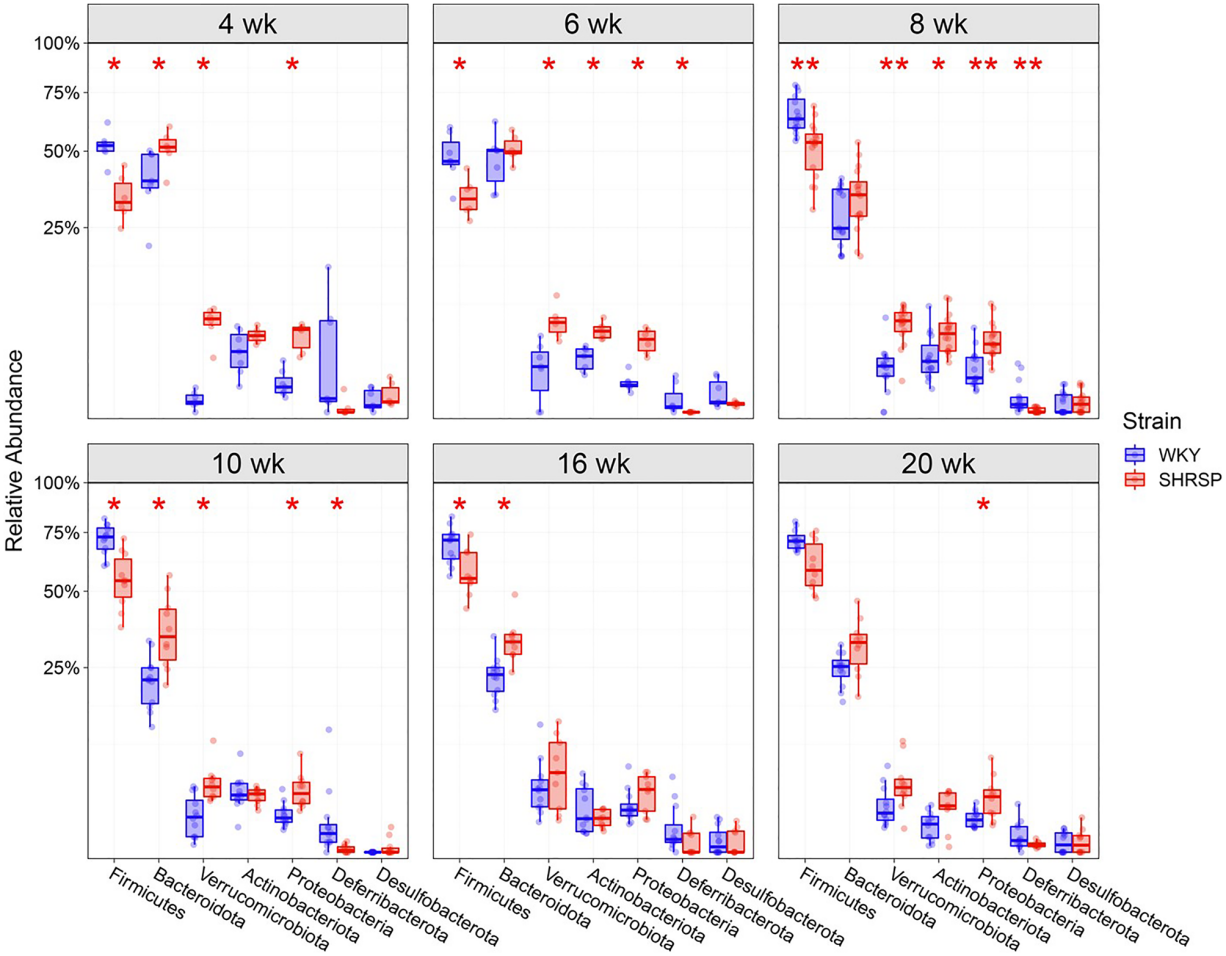
Relative abundance of phyla in SHRSP and WKY at 4, 6, 8, 10, 16 and 20 weeks (n = 6–15). * and **p < 0.05 and 0.01 respectively compared to WKY at the same age using Mann–Whitney U test with FDR corrected for multiple comparisons.

Relative abundance of bacteria phyla at different ages are shown in Fig. 3. The relative abundance of Firmicutes was greater in WKY compared to SHRSP; however, statistical significance was not achieved at 20 weeks. On the other hand, abundance of Bacteroidota was greater in SHRSP with significance being achieved at 4, 10 and 16 weeks of age. Overall, there was a contraction of Firmicutes and expansion of Bacteroidota, Verrucomicrobiota, and Proteobacteria in SHRSPs compared to WKYs.

**Figure 4:**
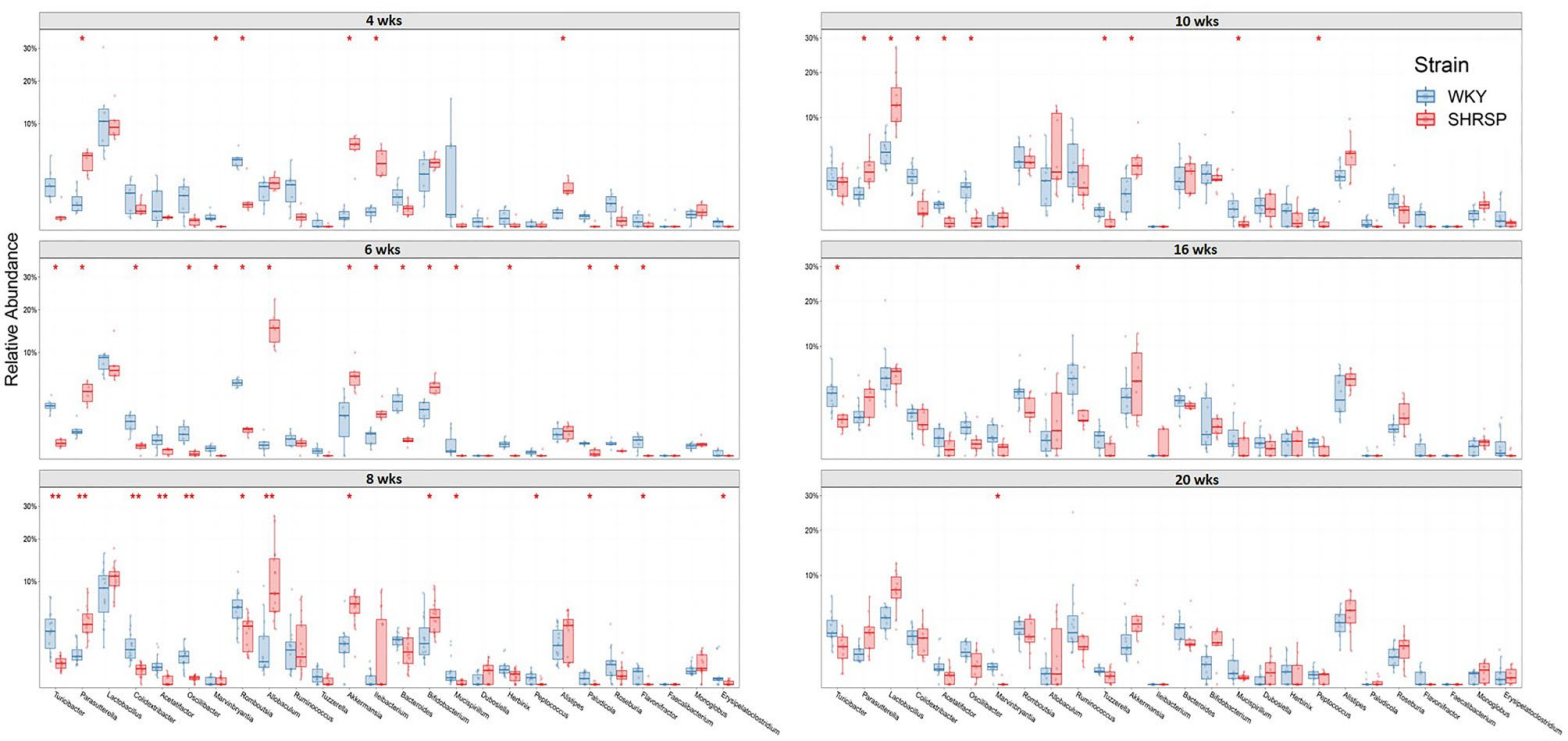
Relative abundance of genera in SHSRP and WKY at 4, 6, 8, 10, 16 and 20 weeks (n = 6–15). * and ** *p* < 0.05 and 0.01 respectively compared to WKY at the same age by Mann–Whitney U test with FDR corrected for multiple comparisons.

Relative abundances at the genus level show a number of differences between WKYs and SHRSPs at different ages (Fig. 4). Note that Fig. 4 presents a portion of the genera identified; genera of very low abundance and genera that did not reach statistical significance were omitted. There was a total of 80 classified and unclassified genera identified in fecal samples from at least one age group. A number of genera were consistently different between strains with age. For example, *Akkermansia* was increased or trended to increase in SHRSP in most age groups with the greatest increases occurring in the younger cohorts. *Akkermansia* was < 1% in WKYs and was significantly increased to 6% in SHRSP at 4 weeks, increased from 1 to 6% at 6 and 8 weeks, and from 1 to 4% at 10 weeks (Fig. 4). Although abundance of Akkermansia increased at 16, and 20 weeks, it was not statistically significant.

*Allobaculum* was significantly increased in SHRSPs at 6 and 8 weeks compared to WKYs with the increases being < 1–16% and 1–8%, respectively (*p* < 0.05, Fig. 4). *Parasutterella* increased or trended to increase in SHRSP at all ages. At 4, 6, 8, and 10 weeks, *Parasutterella* significantly increased (*p* < 0.05 for each), at 4 weeks < 1–4%, 6 weeks < 1–4%, 8 weeks 1–3%, and 10 weeks < 1–3%.

*Turicibacter* significantly decreased (*p* < 0.05 for each) in SHRSP at 4 weeks 2% to < 1%, 6 weeks 2% to < 1%, 8 weeks 2 to < 1%, and 16 weeks 3% to < 1%. *Oscillibacter* showed a similar trend of decreasing in SHRSP.

**Figure 5:**
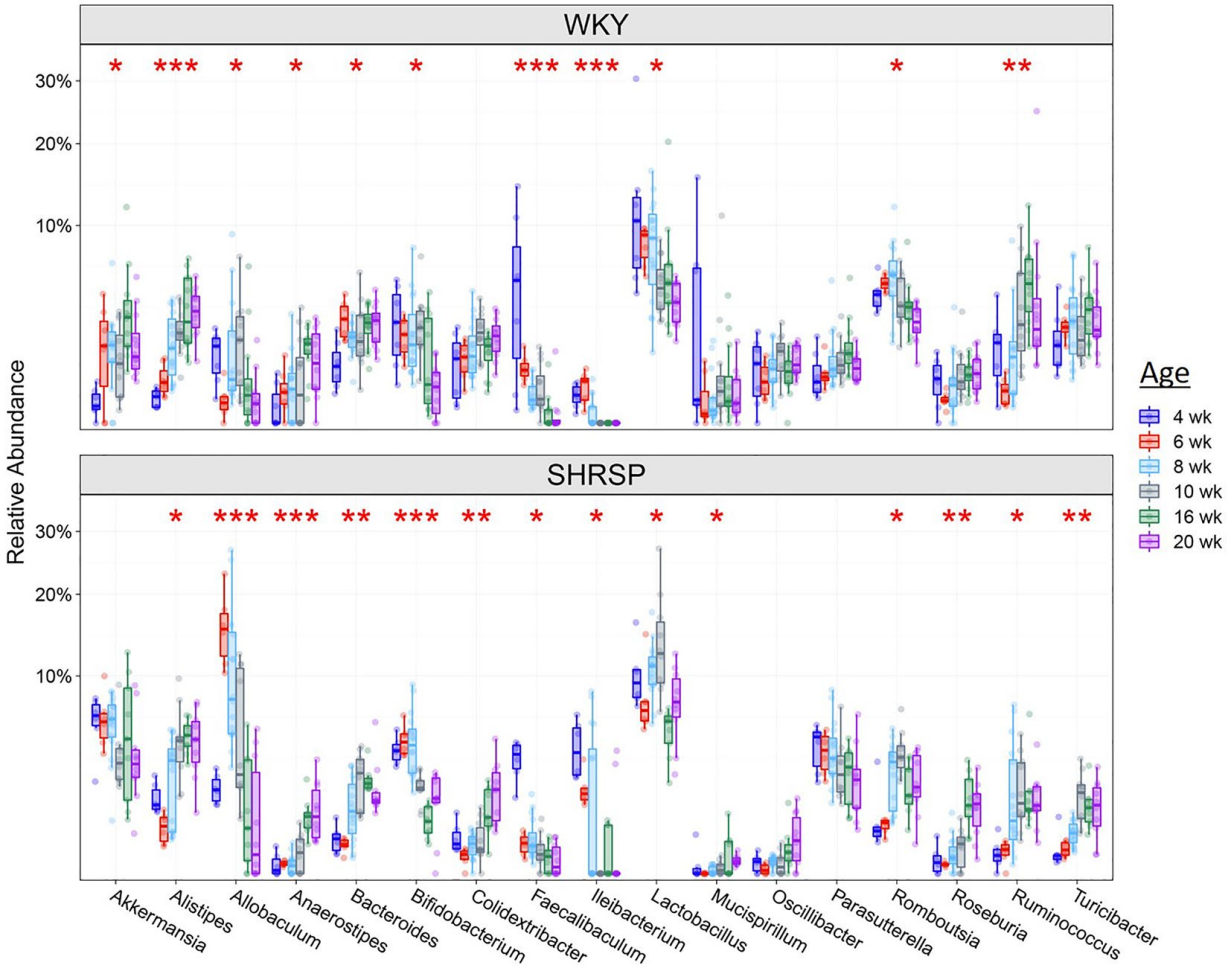
Relative abundance of genera in WKY (top) and SHRSP (bottom) during aging from 4 to 20 weeks. (n = 6–15) *, **, and ***p < 0.05, 0.01, and 0.001 over age respectively using Mann–Whitney U test with FDR corrected for multiple comparisons. Only genera with relative abundance ≥ 0.5% are shown.

After comparing community structures between SHRSPs and WKYs at different ages, we evaluated changes in community structure in individual strains as the rats aged. A change in SHRSP bacterial community could represent an age where a phenotypic expression such as SBP or BBB integrity would be altered as a result of the altered gut microbiota. PCoAs of weighted UniFrac in individual strains as the rat aged are shown in Supplemental Figure I. SHRSP showed significantly different microbiota structures as assessed by weighted UniFrac distance over the ages of 4–20 weeks (*p* = 0.001). Similarly the weighted UniFrac distance was significantly different in WKYs from 4 to 20 weeks (*p* = 0.001). For both strains, the 4 and 6 weeks communities segregated from that of all other ages. Furthermore, this shift can be seen when looking at the genera abundance (Fig. 5) where a number of genera showed increases or decreases in abundance between 6 and 10 weeks. Thus, if a shift in community structure is responsible for the increase in SBP, it is likely to have occurred between 6 and 10 weeks, ages when SBP showed initial increases (Fig. 1).

**Figure 6:**
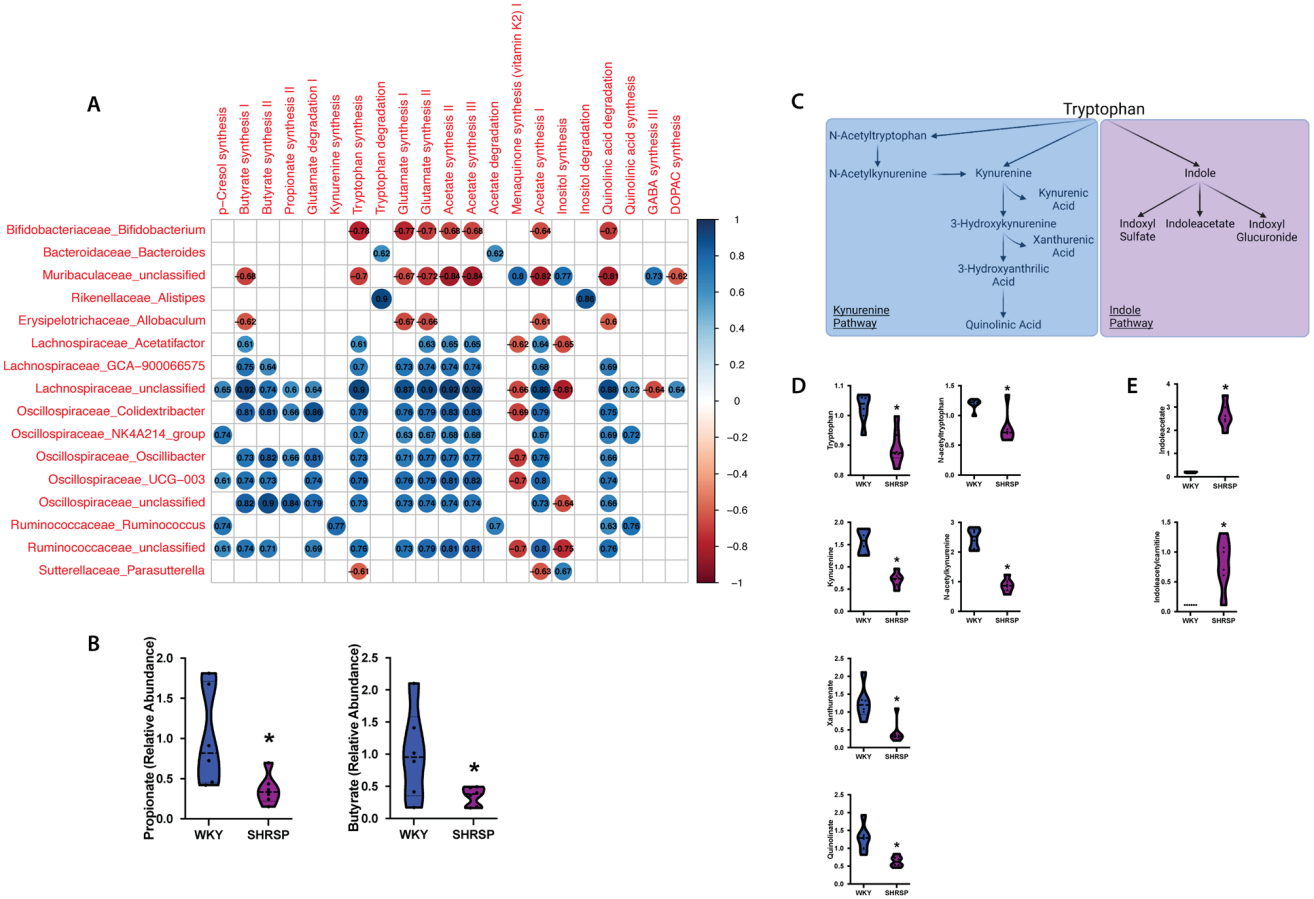
(**A**) Heatmap of Spearman correlations between gut-brain modules (GBM) and genera relative abundance. Genus-GBM pairs with correlations >  = 0.6 or <  = −0.6 and *p*-value < 0.05 are shown. (**B**) Short-chain fatty acid measurement of WKY and SHRSP plasma. (**C**) Schematic diagram of tryptophan metabolism pathways. D-E. WKY and SHRSP plasma metabolites of kynurenine pathway (**D**) and indole pathway (**E**). (n = 6) *p < 0.05 using Mann–Whitney U test with FDR corrected for multiple comparisons.

Next, we aimed to examine the functional capacity (metabolites and/or biochemical pathways) of WKY and SHRSP gut microbiota. The functional capacity of WKY and SHRSP microbial communities across all ages was inferred using Phylogenetic Investigation of Communities by Reconstruction of Unobserved States (PICRUSt2). From 16S rRNA sequencing data, PICRUSt2 was used to generate a list of genes predicted in each community. The gene lists were used to calculate a predicted abundance of functional modules involved in gut-brain communication (gut-brain modules; GBM)^23^. Fig. 6A shows the correlation scores between GBM abundance and genera abundance. We observed several strong positive correlations between genera abundance and short-chain fatty acid (SCFA) and amino acid (AA) metabolism modules. To validate the predicted differences in SCFA and AA metabolism we performed targeted and untargeted metabolomics, respectively, in plasma of SHRSP with established HT and WKY. We observed significant reductions in propionate and butyrate concentrations in SHRSP plasma (Fig. 6B). We performed random forest classification to identify metabolites important in distinguishing WKY from SHRSP^13^. We found tryptophan metabolism to be a strong predictor of WKY versus SHRSP genotype^13^. Examining individual metabolites, we found significant increases in metabolites along the kynurenine pathway in WKY plasma, including kynurenine, xanthurenate, quinolinate, N-acetyltryptophan, and N-acetylkynurenine (Fig. 6D). In SHRSP plasma we observed significant increases in metabolites along the indole metabolism pathway, including indoleacetate and indoleacetylcarnitine (Fig. 6E). Bacterial enzymes are capable of converting tryptophan to kynurenine or indole. Our findings suggest the dysbiosis in SHRSP may lead to alterations in tryptophan metabolism and indicate that further investigation into tryptophan metabolites as a means of microbe-host interaction in the context of HT is warranted.

## Discussion

Solid evidence supports the idea that gut dysbiosis is sufficient to elicit HT in the SHRSP. Conversely, prevention of dysbiosis attenuates HT^12,13,24^. These findings in the SHRSP model of HT support the idea of a potential role for gut dysbiosis in the development of HT in humans and other animal models and also demonstrates the importance of the gut microbiota, in general, in the development and maintenance of pathological states^7–13,25^.

Given the importance of the gut microbiota in pathological states, we sought to identify the taxa responsible for the HT and determine when those taxa were altered in the SHRSP microbiota. We report three main findings in the present study. (1) The microbiota community structure in WKY and SHRSP were significantly different as early as 4 weeks of age and remained significantly different for the remainder of the study (Fig. 2). (2) Given the number of genera that were different between WKY and SHRSP at ages when HT was developing, it is possible that an overall change in the microbial community structure, as opposed to a change in a single or a few bacteria, was responsible the development of HT (Fig. 4). (3) By assessing the functional capacity of the microbial communities and examining microbial metabolites in plasma, we identify reduced short chain fatty acids and increased shunting of tryptophan to indole in the SHRSP (Fig. 6). Each of these findings will be discussed in more detail below.

1. The microbiota structures in WKYs and SHRSPs were significantly different at 4 weeks of age and remained significantly different for the remainder of the study (Fig. 2). This is in line with a previous study that showed significant differences between the WKY and SHR microbiota community structure as early as 1 week old^26^. We observed differences in taxa abundance at the phylum and genus levels (Figs. 3 and 4); however, given the large number of genera that were significantly different between WKY and SHRSP over time, it was impractical to determine an individual genus or group of genera that was responsible for the HT. The microbiota component of HT in SHRSP was likely driven by an overall change in the community and not by a single genus or a small group of genera.
2. We observed changes in community structure as WKY and SHRSP aged; however, no age-related changes in the gut microbiota could readily be identified as a causal factor for the development of HT in SHRSP. The community structure as measured by the weighted UniFrac distance showed a clear segregation at 4 and 6 weeks in both WKY and SHRSP when compared to all other age groups (Supplemental Figure I). It is likely that these early difference in community structure was important to the phenotype that developed in later life for both strains of rats^26^. Changes in community structure in the microbiota of SHRSP subsequent to the development of HT may be secondary to, or responsible for maintaining, the HT.
3. Given the difficulty in trying to identify a single genus or small group of genera responsible for the SHRSP phenotype, we turned to examine differences in the overall functional capacity of the microbial communities. Using 16S rRNA sequencing data, PICRUSt2 was used to quantify gene abundance in a community based on sequenced bacterial genomes. We focused on gene pathways that are characterized by an involvement in gut-brain communication. We found several strong positive correlations between the microbiota and gene pathways involved in SCFA and amino acid metabolism (Fig. 6A). We further explored each of these pathways by measuring metabolites in the plasma. Similar to previous observations in the SHR model, we observed significant reductions in plasma propionate and butyrate of SHRSP, relative to WKY (Fig. 6B). Impaired SCFA receptor signaling in the gut, vasculature, and kidney has previously been linked to HT^14,17,27^.
  Bacteria found in the gut possess the enzymes for converting tryptophan to indole or kynurenine as well as many of the downstream metabolites^28^. We observed a number of tryptophan derived metabolites to be significantly different between WKY and SHRSP. Tryptophan can be converted to serotonin, enter the kynurenine pathway, or the indole pathway (Fig. 6C). We found that a number of metabolites in the kynurenine pathway were significantly elevated in WKY relative to SHRSP plasma. Conversely, indoleacetate and indoleacetylcarnitine, metabolites of indole metabolism, were elevated in SHRSP plasma (Figs. 6D and E). These data suggest the dysbiotic SHRSP microbiota preferentially shunts tryptophan to the indole pathway resulting in elevated indole metabolites and decreased kynurenine metabolites. Increased indole production can lead to the accumulation of uremic toxins, including indoxyl sulfate, p-cresyl glucuronide, and p-cresyl sulfate, which are pro-hypertensive through their inflammatory and oxidative effects in peripheral tissues such as the vasculature and kidney^29^. However, other indole metabolites negatively correlate with BP. For example, increased salt intake elevates BP while reducing *Lactobacillus murinis*, indole-3-acetic-acid, and indole-3-lactic-acid^30^. The disparate effects of various indole metabolites on BP may be secondary to the presence of multiple indole receptors distributed on a wide range of cell types, including epithelium, endothelium, and innate and adaptive immune cells^31^. Further studies will be required to determine the bacteria involved in altered tryptophan metabolism of SHRSP and how these metabolites may contribute to the hypertensive phenotype.

Overall, these studies provide further evidence that the dysbiotic microbiota of SHRSP contributes to the hypertensive phenotype of this model. A specific genus, or group of genera, could not be determined as responsible for the observed elevation in BP. Rather, our data support the idea that an overall change in the microbiota structure and microbial metabolites leads to HT. In support of this, we identify significant reductions in circulating propionate and butyrate, as well alterations in tryptophan metabolites that suggest preferential shunting of tryptophan to indole metabolism in the SHRSP model.

## Supplementary Information

Supplementary Information 1.

Supplementary Information 2.

## Data Availability

The datasets generated during the current study are available in the GenBank repository, accession KFUL00000000. Additional sample metadata are available from the corresponding author on reasonable request.
