# Supplementary figures and images for: Alterations of the gut microbial community structure and function with aging in the spontaneously hypertensive stroke prone rat
Source: Sci Rep. 2022 May 20;12:8534. doi: 10.1038/s41598-022-12578-7 (PMC9122926; doi:10.1038/s41598-022-12578-7)

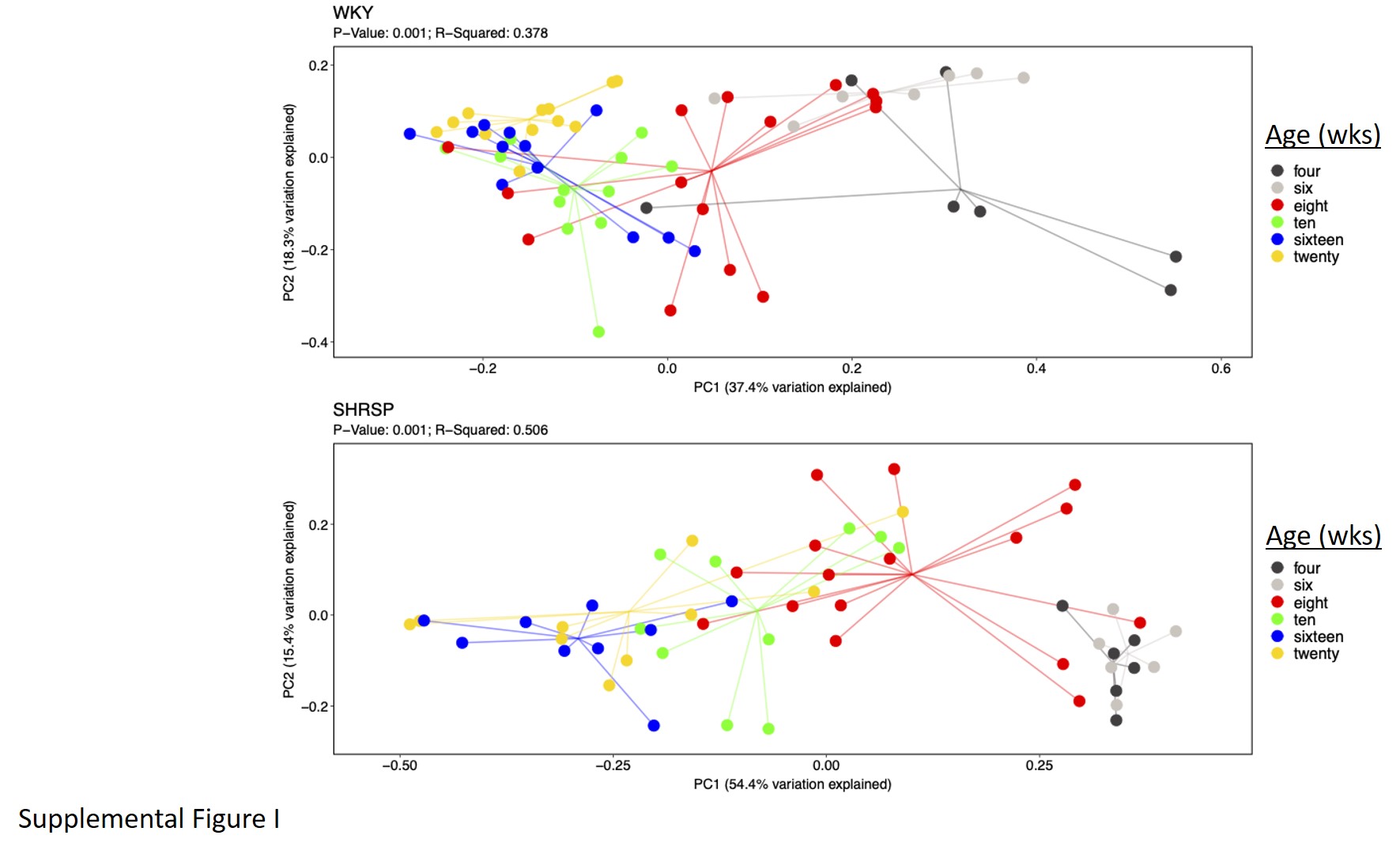

Supplement: Supplementary file 1 — Supplementary Information 1. [file 41598_2022_12578_MOESM1_ESM.jpg]
