# Supplementary material for: Alterations of the gut microbial community structure and function with aging in the spontaneously hypertensive stroke prone rat
Source: Sci Rep. 2022 May 20;12:8534. doi: 10.1038/s41598-022-12578-7 (PMC9122926; doi:10.1038/s41598-022-12578-7)
Supplement: Supplementary file 2 — Supplementary Information 2. [file 41598_2022_12578_MOESM2_ESM.docx]

Supplemental Figure I. PCoAs of weighted UniFrac at 4, 6, 8, 10, 16, and 20, weeks in WKY (top) and SHRSP (bottom) (n=6-15). P-values, as determined using permutational multivariate analysis of variance (PERMANOVA) are at the top of each plot.
